# Lipid Oxidation Effect on Membrane Properties in Phosphatidylinositol- and Phosphatidylserine-Enriched Neuronal Biomimetic Models

**DOI:** 10.1021/acs.jpcb.6c00805

**Published:** 2026-07-23

**Authors:** Dominik Drabik

**Affiliations:** † Department of Biomedical Engineering, Wroclaw University of Science and Technology, Wroclaw 50-370, Poland; ‡ Department of Cytobiochemistry, Faculty of Biotechnology, University of Wroclaw, Wroclaw 50-383, Poland

## Abstract

Oxidative stress is a key driver of neurodegenerative diseases, yet its impact on the mechanical properties of complex neuronal membranes remains poorly defined. A combined computational and experimental bottom-up approach was employed to study biomimetic membranes, ranging from simple bilayers to complex mixtures mimicking human neuronal composition. The results indicated that oxidation influenced all investigated membrane properties. While a decrease in membrane thickness and area compressibility was observed across all systems, trends for bending rigidity, area per lipid, and diffusion coefficients remained inconsistent in the simpler models. A significant influence of phosphatidylinositol and cholesterol on these properties was identified. Experimental giant unilamellar vesicles (GUVs) exhibited oxidation levels between 2% and 17%, with cholesterol-rich mixtures demonstrating higher susceptibility to peroxidation. Furthermore, an approach is proposed that demonstrates that deviations in experimentally obtained bending rigidity can be partially explained by the level of oxidation. This study emphasizes that accounting for oxidation is crucial for accurately modeling the mechanical behavior of neurological membranes.

## Graphical abstract

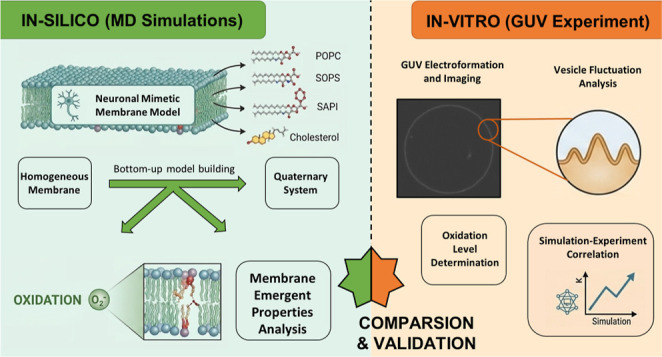

## 1 Introduction

Oxidative stress is slowly growing to the rank of a civilization disease. It often results from a poor diet, consumption of highly processed food, excessive use of stimulants, and prolonged stress. One of the consequences of uncontrolled oxidative stress, i.e., the lack of a balance between the level of oxidants and antioxidants, is damage to cells, tissues, and organs caused by oxidative damage. The brain, with its high oxygen consumption and lipid-rich content, is highly susceptible to oxidative stress.^1^ Excessive damage to biomolecules due to oxidation stress leads to cellular malfunction and neurodegeneration.^2,3^ Several neurological disorders, including Alzheimer’s, Parkinson’s, Amyotrophic lateral sclerosis, multiple sclerosis, and ischemic stroke, are associated with oxidative stress. Interestingly, cell membranes in brain constitute greater than 66% phospholipids by mass,^4^ indicating that the oxidative stress effect on membranes should be systematically investigated.

As the basic building blocks of cell membranes are lipid molecules, it is justified to investigate how they are affected by oxidation stress. The oxidation of lipids is a complex process in which oxidants (such as free radicals or reactive oxygen species) attack lipid molecules containing carbon double bonds (unsaturated).^5^ This leads to the degeneration of acyl chains of lipids (commonly known as rancidity), disruption of their functions, and the formation of peroxide and hydroperoxide derivatives. There exist several studies that focus on the effect of oxidation of lipids on the behavior and properties of lipid membrane models.^6–10^ Phenomena such as the increase in membrane surface area, decrease of membrane thickness, promotion of lipid domain formation, change in lipid order, and formation of transient pores affecting membrane permeability were observed. However, the membrane lipid composition is tightly regulated by the cell, maintaining a homeostasis that, if disrupted, can impair cell function and lead to disease. Furthermore, oxidative stress might also indirectly influence the membrane composition and homeostasis. For instance, the increased oxidative stress in the hippocampus has led to an increase in the amount/activity of sphingomyelinase and thus an increase in the production of ceramides.^11^ It was even hypothesized that it can affect cell delivery and affect the function of membrane peptides.^12^ As a result, simple lipid models, composed of one or two lipid molecules, might not be sufficient to grasp the in-depth effect of oxidation on the membranes itself. Fortunately, the recent development of lipidomics allows the determination of detailed lipid composition and hence allowed the emergence of biomimetic lipid membranes.^13,14^

In this article, such an approach was used. Specifically, the composition of the membrane investigated in this study was based on ratios presented for human brain tissue, superficially an inner membrane of human neurons.^15^ However, a bottom-up approach was used: starting from a simple homogeneous membrane, additional lipid types are added and the impact on membrane properties is investigated. Combined computational and experimental studies of lipid membranes were carried out to determine the change of membrane properties due to the presence of oxidized lipid molecules.

## 2 Materials and Methods

### 2.1 Materials

Lipids POPC (PC­(16:0/18:1­(9*Z*))), SOPS (PS­(18:0/18:1­(9*Z*))), SAPI (PI­(18:0/20:4­(5*Z*,8*Z*,11*Z*,14*Z*))), and cholesterol were purchased from Avanti Polar Lipids (Alabaster, AL, USA). See Figure S1 for the chemical structures of lipids used in the study. The fluorescent probe DiLC was purchased from Atto-Tech (Siegen, Germany). The Lipid Peroxidation (MDA) Assay Kit was purchased from Sigma-Aldrich. Glacial acetic acid, 1-butanol, sulfuric acid, ascorbic acid, and ammonium molybdate were purchased from Sigma-Aldrich. The ultrapure water used in experiments was obtained from a water purification system (Milipore).

### 2.2 Molecular Dynamics Simulations and Parametrization

The full-atomistic MD simulation was performed using NAMD 2.13 ^16^ software with CHARMM36 force fields^17^ under *NPT* conditions (constant: number of particles, pressure, and temperature). Lipid membrane systems consisted of 648 lipid molecules (324 on each of leaflets). The composition of base 6 systems was based on lipidomic data.^15^ The following systems were simulated—POPC/SOPS 80:20, POPC/SAPI 88:12, POPC/SOPS/SAPI 71:19:10, POPC/Chol/SOPS 40:50:10, POPC/Chol/SAPI 42:52:6, and POPC/Chol/SOPS/SAPI 38:47:10:5. Additionally, each system was oxidized to 10% (by randomly selecting lipids to oxidize). Furthermore, the POPC system was also oxidized. This resulted in a total of 13 systems. The detailed composition of each system as well as details of parametrization of oxidized lipids is presented in Supporting Information Table S1. Details for parametrization of oxidized lipid molecules are described in Supporting Information Section 3. Each system was hydrated in such a way that 75 water molecules per lipid molecule were used. All systems were additionally neutralized with positive counterions to maintain agreement with the buffer behavior used in experiments. All systems were equilibrated using the CHARMM standard equilibration procedure.^18^ Total simulation time for all systems was at least 110 ns, with the last 10 ns used for analysis. Simulations were carried at 25 °C (298.15 K) to maintain agreement with experimental data. For each system, three replicas were simulated.

### 2.3 Membrane System Characteristics

#### 2.3.1 Area per Lipid and Membrane Thickness

Both membrane area per lipid (APL) and membrane thickness (MT) were determined using a custom MATLAB script. The midpoint position between P and C2 atoms was used as a point position for each lipid. This was followed by Voronoi tessellation to obtain individual APLs for each lipid molecule in every simulation time step. The APL value for the whole membrane was determined by Gaussian fitting to histogrammed APL values of all molecules. The APL value for a given type of molecule was determined by averaging. MT was calculated as a difference between mean height values of atoms in opposite leaflets. Parameters were determined for P, C1, and C2 atoms. For cholesterol, corresponding C3, C2, and C1 atoms were selected.

#### 2.3.2 Bending Rigidity and Tilt Rigidity

To determine mechanical parameters (focusing on bending rigidity coefficient, but also tilt modulus), a real-space fluctuation (RSF) method was used.^19^ Specifically, a probability distribution for both tilt and splay was determined for all lipids over all analyzed time steps. Tilt θ is defined as an angle between the lipid director (vector between the lipid head—midpoint between C2 and P atoms—and lipid tail—midpoint between last carbon atoms) and bilayer normal. Lipid splay *S*_r_ is defined as the divergence of an angle formed by the directors of neighboring lipids provided that they are weakly correlated.

#### 2.3.3 Compressibility

The methodology established by Doktorova et al.^20^ was used. Unlike traditional approaches that rely on membrane area fluctuations, this method utilizes thickness fluctuations (*t*) to calculate the *K*_A_ values of individual leaflets. Briefly, by applying a combination of thermodynamic and statistical principles, we derived *K*_A_ from an analytical expression incorporating the area per lipid, equilibrium membrane thickness, instantaneous thickness, and thermal energy (*k*_B_ *T*). The computational procedure involves an initial optimization of the membrane thickness layer to prevent the over- or underestimation of the *K*_A_. This is followed by the binning of local fluctuations to determine the potential of the mean force (PMF). Finally, the *K*_A_ for each leaflet is calculated based on the relative changes in the membrane thickness.

#### 2.3.4 Lateral Diffusion Coefficient

The Diffusion Coefficient Tool plugin was used for lipid molecules lateral (2D) diffusion determination.^21^ It is calculated using Einstein’s relation with mean square displacement (MSD) of the chosen molecular species.

#### 2.3.5 Interdigitation

The interdigitation parameter was determined using MEMBPLUGIN.^22^ The obtained parameter is defined as the width of the region of mass overlap.

### 2.4 GUVs Electroformation

The electroformation method of model membrane formation for giant unilamellar vesicles (GUVs) was used.^23^ Briefly, 10 μL of a 1 mM lipid mixture and a fluorescent probe mixture (1m %) in chloroform was distributed equally along the platinum electrodes and dried under reduced pressure for 1 h. The electrodes were then submerged in aqueous nonconductive solution, and a sinusoidal 10 Hz AC electric field was applied for 4 h with 1 V voltage in a custom PTFE (polytetrafluoroethylene) electro-formation chamber.

### 2.5 Flicker Noise Spectroscopy

Thermally induced shape fluctuations of GUV can be used to determine the mechanical properties (bending rigidity). A series of images of GUVs were recorded using confocal microscopy. A Stellaris 8 confocal microscope (Leica, Germany) was equipped with an HC PL APO 86×/1.20 water immersion objective (Leica, Germany) and 1 Airy unit pinhole size. 256 × 256 pixels images were recorded with a hybrid (HyD) detector with pixel size ranging from 0.07 to 0.12 μm with video integration time ranging from 148 ms up to 189 ms depending on the zoom magnitude. Samples were illuminated with a white laser set at 560 nm—emitted light was recorded from 570 up to 630 nm. The series usually consisted of 1200 images. The two-dimensional liposome images are transformed to the three-dimensional Helfrich model using a statistical approach. Specifically, the radial position of the bilayer, extracted from images, is used to construct angular autocorrelation curves. The curves are decomposed into Fourier series, and a frequency histogram of amplitudes for each mode of fluctuation is calculated. The histogram is then used for determination of the bending rigidity coefficient as described in detail elsewhere.^24^

### 2.6 Oxidation Level Assessment

GUVs (1500 μL) were divided into two samples (700 μL). To assess concentration of lipid, the ascorbic acid + molybdate method was used.^25^ Solutions of KH_2_PO_4_, corresponding to 0, 0.5, 1, 1.5, 2, 2.5, and 5 μg of phosphorus, were dissolved in 50 μL of water in glass vials. Subsequently, 0.5 mL of concentrated perchloric acid (70%) was added to each vial, and the mixtures were heated to 200 °C for 2 h under cover to achieve phosphate mineralization. After cooling, 1 mL of an aqueous solution containing 2.5 wt % ammonium molybdate and 10 wt % ascorbic acid was added to each vial, followed by vortexing. The samples were then incubated at 37 °C for 1 h. Next, the samples were transferred to a 96-well plate, ensuring that each sample was distributed into at least three wells. After further cooling, absorbance was measured at 800 nm using an Asys UVM340 Plate Reader (Biochrom). GUV samples (750 μL) were evaporated overnight at 95 °C and subsequently resuspended in 50 μL of water to match the conditions used for calibration standards. These samples were then processed using the same protocol described above. Finally, the lipid mass was estimated based on the calibration curve and the measured absorbance values of the GUV samples.

To assess the oxidation level, the following protocol was used. 150 μL of concentrated sulfuric acid was added to 750 μL of GUV solution. Subsequently, the solution was vortexed, and 300 μL of TBA (suspended in glacial acetic acid and water 3:7) was added. It was vortexed and was heated for an hour in 95 °C and then thawed on ice. This was followed by the addition of 500 μL of 1-butanol and vortexing. The solution was then centrifuged in 16,000*g* for 10 min. Then, the 1-butanol layer was transferred to another vial and evaporated at 100 °C. After evaporation and cooling, the remaining material was resuspended in 200 μL of water, vortexed, and transferred to a 96-well plate. The absorbance was measured at 532 nm. Reference points were prepared according to the kit manual.

### 2.7 Statistics

In order to test for the significant difference between the parameters, unless specified otherwise, the one-way ANOVA test was used with the significance level at 0.05. The Tukey test was used as a post hoc test. All statistical analyses were performed using OriginPro 2015 (OriginLabs) software. Average values are presented with the standard deviation.

## 3 Results and Discussion

### 3.1 Molecular Dynamic Characterization of Membrane Models

The membrane composition was modeled after the human neuronal plasma inner membrane, as demonstrated by Ingolfsson et al.,^15^ specifically the PC/PE/SM/PS/PI/PA/Chol (14:22:2:10:5:1:46) ratio. To ensure a manageable yet representative model for both computational and experimental analyses, several systematic simplifications were implemented. First, minor components representing the lowest abundance in the native membrane—specifically sphingomyelin and phosphatidic acid—were excluded to reduce complexity of the system. Second, phosphatidylethanolamine (PE) was substituted with phosphatidylcholine (PC). This substitution was justified by the fact that PC/PE interactions are extensively documented in the existing literature; by consolidating these fractions into a single zwitterionic PC component, it was possible to focus more precisely on the specific influences of the remaining molecules. The rationale for the current model was to prioritize the diversity of lipid head-groups while maintaining the number of lipid species at a manageable level for both the simulation and experiment. Consequently, this approach entails a simplified representation of the complex acyl chain distributions typically found in native neuronal membranes. As a result, the proposed lipid membrane model used in this study is composed of 4 molecule types—POPC/Chol/SOPS/SAPI 38:47:10:5. However, as the bottom-up approach was implemented, all viable mixtures (those that are able to form vesicles in vitro) were also investigated. These systems were subjected to detailed characterization of their topological and mechanical properties. Furthermore, in each system, 10% of lipids were selected randomly and oxidized to investigate the effect of oxidation on those properties. To ensure consistency between the simulation and experimental data, specific simplifications to the oxidation process were introduced. Oxidation products were limited to aldehyde-bearing truncated lipid molecules to align with the experimental methodology for oxidation determination (TBA assay), and cholesterol oxidation was not considered. Additionally, the oxidation of SAPI species was restricted to the second double bond; however, given the low concentration of these species within the system, it is improbable that varying the truncation site would yield statistically significant changes in global membrane properties. The change between the system prior to and after oxidation is presented in Table 1, while the individual parameters of each of the systems are presented in Table S1.

**Table 1:** Change in Investigated Membrane Properties before and after Oxidation^a^ ^,^ ^b^

Lipid Membrane	ΔMT_P–P_ [nm]	ΔAPL [Å^2^]	Δκ [10^–19^ J]	Δκ_tilt_ [10^–20^ J]	Δ*K*_A_ [mN/m]	Δ*D* [μm^2^/s]
POPC	(−0.18 + 0.01)	(−0.77 + 0.22)	(0.21 + 0.02)	(−0.4 + 0.1)	(−56 + 52)	(0.11 + 0.14)
POPC/SOPS	(−0.51 + 0.03)	(9.1 + 1.1)	(0.16 + 0.11)	(−0.1 + 0.3)	(−432 + 51)	(−0.03 + 0.08)
POPC/SAPI	(−0.14 + 0.03)	(0.6 + 0.8)	(−0.38 + 0.11)	(−0.5 + 0.3)	(−291 + 26)	(0.48 + 0.68)
POPC/SOPS/SAPI	(−0.11 + 0.03)	(−2.5 + 4.5)	(−0.07 + 0.06)	(−0.4 + 0.3)	(−134 + 57)	(0.32 + 0.66)
POPC/Chol/SOPS	(−0.5 + 0.3)	(−1.0 + 0.6)	(−0.15 + 0.02)	(−0.8 + 0.9)	(−3941 + 325)	(0.07 + 0.51)
POPC/Chol/SAPI	(−0.22 + 0.17)	(−0.8 + 0.2)	(−0.15 + 0.07)	(−5.4 + 2.3)	(−7533 + 160)	(0.1 + 0.3)
POPC/Chol/SOPS/SAPI	(−0.08 + 0.07)	(−1.9 + 1.2)	(−0.90 + 0.04)	(−12 + 1)	(−6572 + 165)	(0.03 + 0.05)

a Abbreviations: MT—membrane thickness, APL—area per lipid, κ—bending rigidity, κ_tilt_—tilt rigidity, *K*_A_—area compressibility, *D*—diffusion coefficient.

b Parameters for POPC were taken from refs 26 and 27 .

A decrease in MT value after oxidation was observed in all of the investigated cases. It was most strong in the case of POPC/SOPS and POPC/Chol/SOPS lipid membranes. Interestingly, the presence of cholesterol in the membrane did mitigate the phenomena in all of the investigated membranes. The observed decrease after oxidation was also observed in both Makky and Tanaka^28^ and Bagheri et al.^8^ in POPC membranes after oxidation. However, in Schumann-Gillet and O’Mara,^29^ no significant changes to MT were observed after oxidation. There was no specific trend regarding average area per lipid (APL) of membrane change after oxidation. In the case of the homogeneous POPC membrane, the APL decreased with oxidation. The decrease is in agreement with literature reports.^8,29^ On the other hand, in POPC membranes with either SOPS or with SAPI the APL increased after oxidation (especially strong change was observed in POPC/SOPS). It should be also noted that the change is lower than the uncertainty value, with the exception of POPC/SOPS. This effect, again for the POPC membrane, was also reported by Wiczew et al.^6^ The reasoning for the APL increase might be that the acyl-chain with oxygen atoms moves over the phosphorus layer and hence increases the area for the head.^30^ In all cases, the presence of cholesterol reversed the phenomenon, showing a slight decrease of APL. This decrease in membranes with cholesterol after oxidation was shown for the POPC/Chol system.^29^ The unspecific relation might be due to the fact that each oxidation product can have a different effect over membrane properties, as stated by Oliveira et al.^31^ The Voronoi graphs for APL determination for oxidized systems are presented in Figure S2.

Furthermore, an unspecific trend was also observed in the case of mechanical property—bending rigidity. In the case of homogeneous POPC and POPC/SOPS, a small increase of bending rigidity was observed after oxidation. However, in the case of POPC/SAPI, a reverse change was observed, and the combination of those two lipids in the POPC/SOPS/SAPI membrane resulted in only a small decrease after the oxidation. In the literature, a decrease of bending modulus after oxidation is commonly reported; a reduction in bending modus was observed in the POPC membrane after oxidation.^32^ In another work,^33^ where PLPC and PDPC lipids were used, a potential reduction in bending modulus could be observed as the bilayer became mechanically more fragile. However, these reports mostly relate to PC or polyunsaturated fatty acid (such as SAPI) membranes. Interestingly, the presence of cholesterol in each of those membranes made the change stronger and unified the tendency; the bending decreased in all membrane systems with cholesterol. Surprisingly, the decrease in POPC/Chol/SOPS/SAPI was stronger than the simple summation of analogous systems with SOPS and SAPI. While it was reported that oxidation does decrease mechanical stability^34^ and reduces membrane mechanical resistance,^35^ this results strongly indicates the previous statement that each oxidation product can have a different effect over membrane properties. Interestingly, in the case of red blood cells, an increase in bending rigidity was observed.^36^ In all membranes, a decrease in tilt rigidity was observed after oxidation. Interestingly, it was stronger in the system with cholesterol, indicating a strong reorganization of those membranes. Assuming an interpretation that tilt rigidity is the energy required for the molecule to tilt (the more limited the possibility of tilt of the particles, the greater the tilt modulus), the oxidation limits the tilting of molecules. In all investigated systems, the oxidation decreased the area compressibility coefficient. This is in agreement with the literature as a general trend.^37,38^ The change in the area compressibility after oxidation was more profound in membranes with cholesterol. The cholesterol by itself has a strong stiffening effect on the membrane as it reduces the area per lipid and increases the bilayer thickness.^39^ This tighter packing limits area fluctuations, which directly increase *K*_A_. However, the strong decrease in the *K*_A_ membrane with cholesterol after oxidation was only indicated in a few studies. DPPC/Chol (67.5:32.5) cholesterol bilayers in the presence of aspirin (which can promote oxidative-like disorder) were reported to cause a strong decrease in *K*_A_.^40^ Furthermore, cholesterol-containing vesicles (DOPC/Chol 1:1) were found to be more susceptible to oxidative stress than pure DOPC vesicles.^41^ It can be carefully stated that a simple mechanism is observable here: oxidized lipids have one of their acyl chains truncated and often reorient toward the aqueous phase;^42^ this results in the disruption of tight packing (that is a result of the cholesterol presence). This is consistent with a softer, more deformable membrane as it increased the motional freedom of both oxidized and adjacent nonoxidized acyl chains. The effect is stronger in cholesterol-enriched membranes, which were tightly packed from the start. Finally, the change in diffusion coefficient was also different depending on the lipid membrane mixture, although in neither case was the difference higher than the uncertainty of the parameter determination. It should be noted that membranes with cholesterol exhibited a lower diffusion coefficient than those without (see Table S2). This is unsurprising as cholesterol limits lipid movement.^43,44^ There are a few studies investigating the effect of oxidation of lipid mobility. It was reported with MD studies that diffusion coefficient decreased in a short time, studied in PLPC lipid membranes^34^ as well as in POPC membranes.^45^ This was also observed in Parkkila et al.,^46^ where a decrease in diffusion coefficient (specifically an increase in diffusion time) was observed in monolayers with truncated POPC. On the contrary, increased local and lateral mobility of lipids upon oxidation was observed using fluorescence correlation spectroscopy and EPR spectroscopy.^42^ An increase in diffusion coefficient for POPC and POPC/SOPS membranes, which is contrary to most of the referred research, was observed.

The membrane properties investigated in this study are emergent properties, arising from collective phenomena and cooperative interactions within the lipid population rather than a simple summation of individual molecular behaviors. Nevertheless, these changes can be partially linked to the structural and conformational states of individual lipid molecules. To this end, the order parameter *S*_CD_ was determined for each lipid subpopulation (see Figure S3A–C). In all unoxidized systems, the POPC subpopulation remained relatively constant and was largely indifferent to the presence of SOPS, SAPI, or both. In contrast, significant conformational changes occurred within the SOPS and SAPI fractions. As expected, the presence of cholesterol induced a significant alteration in *S*_CD_ across all investigated subpopulations. Interestingly, in oxidized systems, the conformation of the remaining unoxidized lipids was also modified (see Figure S3D,E), with all lipid species exhibiting shifts in *S*_CD_ values across all carbon atoms. Notably, the *S*_CD_ of SOPS increased significantly from the 10th carbon atom upward. Even the previously indifferent POPC fraction exhibited higher *S*_CD_ values than in the unoxidized state. These results indicate that even in unoxidized systems, there are strong interactions between SOPS and SAPI lipids. Furthermore, the introduction of oxidation affects the entirety of the system and the structural integrity of all constituent lipids; the impact is not localized solely to the immediate vicinity of the oxidative event but propagates throughout the membrane patch.

### 3.2 Characterization of GUVs

In the second stage, the previously characterized lipid mixtures were used to prepare GUV models. Obtained vesicles were used as a model for both determination of bending rigidity coefficient and determination of oxidation level in the samples. Snapshots of POPC/SOPS/SAPI and analysis using a statistical approach are presented in Figure 1A,B. The results of bending rigidity are presented in Figure 1C. As most of the article discusses the bending rigidity change in reference to the POPC membrane, bending rigidity equal to 21.5 *k*_B_ *T* (12 × 10^–20^ J)^26^ was taken as the reference. An increased bending rigidity of POPC/SOPS in reference to POPC was observed. This is supported by the literature as by using micropipette aspiration and X-ray diffraction, it was shown that PS lipids (including SOPS-like species) increase bilayer rigidity due to headgroup interactions.^47^ On the other hand, an increase in bending was also observed in the case of the POPC/SAPI membrane, which is against the indication that SAPI, as well as other polyunsaturated acyl-chains, is identified with low bending rigidity.^48^ The increase of bending rigidity due to the presence of cholesterol is an expected and known phenomenon.^49^ It should be noted that the obtained values of bending rigidity differed slightly from values for not oxidized systems obtained with molecular dynamics. To investigate a possible explanation for this deviation, the oxidation level in the GUVs samples was analyzed.

**Figure 1:**
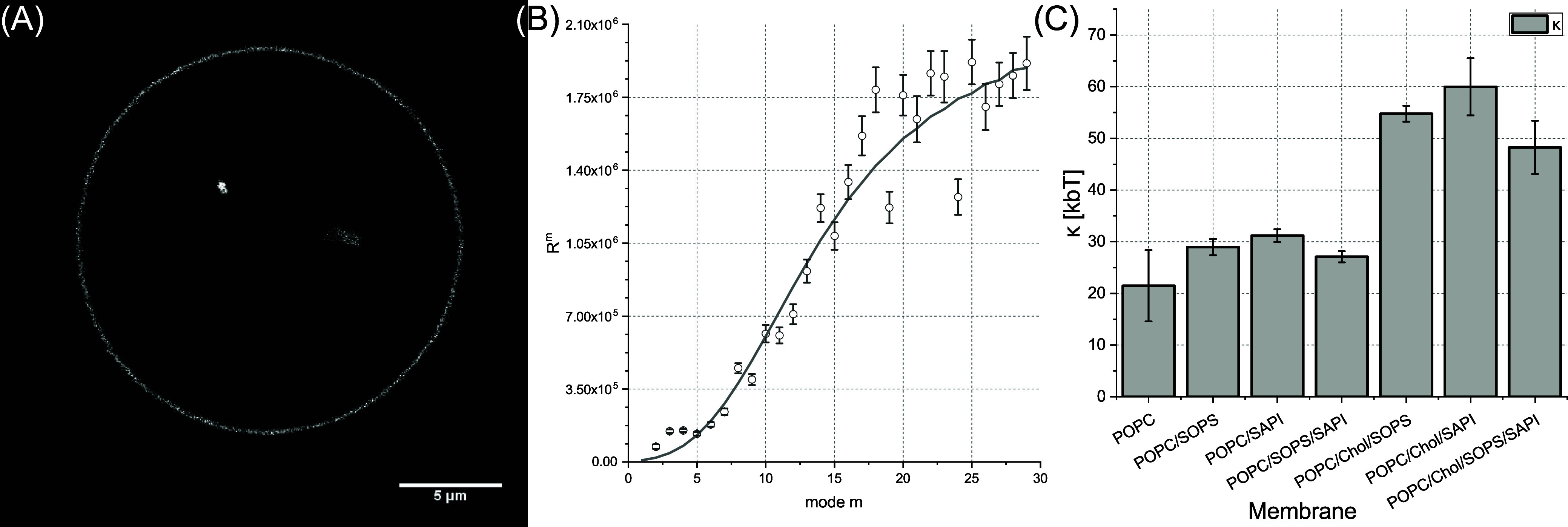
(A) Example image of the POPC/SOPS/SAPI GUV labeled with the 1m % DiLC fluorescent probe. (b) Example of statistical analysis for POPC/SOPS/SAPI used to determine bending rigidity. (c) Obtained values of bending rigidity (κ) for investigated systems. POPC bending rigidity was referenced from ref 26.

The obtained oxidation levels of samples were in the range of 2% up to 17%, depending on the samples, as shown in Figure 2 (detailed information along calibration curves is presented in Figure S4 and Table S2). The lowest oxidation was observed for the homogeneous POPC vesicle (it was indicated that prolonged electroformation can lead to an increase in oxidation,^50^ however, there was no significant change between vesicles electroformed for 2 and 8 h). Furthermore, mixtures with SOPS had higher oxidation than either POPC/SAPI or POPC/SOPS/SAPI mixtures. Additionally, the deviation between individuals was two times higher than in other lipid mixtures. Interestingly, the oxidation level for mixtures with cholesterol was higher than that for those with only phospholipids. The possibility that cholesterol oxidation products would impact the measurements was excluded as those cannot be measured using TBA. This result is in agreement with the literature as it was indicated that cholesterol influences the rate of lipid peroxidation in biological membranes.^51,52^ Alternatively, it is also possible that electroformation induced oxidation at the start of the process (the length of it does not have a significant influence), and though the same amount of lipids was affected, due to fewer lipids in cholesterol mixtures, the oxidation percentage is higher. Nevertheless, due to the observed variation on oxidation percentage of the investigated samples, a question arose: whether the oxidation observed in the in vitro samples can be a sole factor of bending deviation from the simulation.

**Figure 2:**
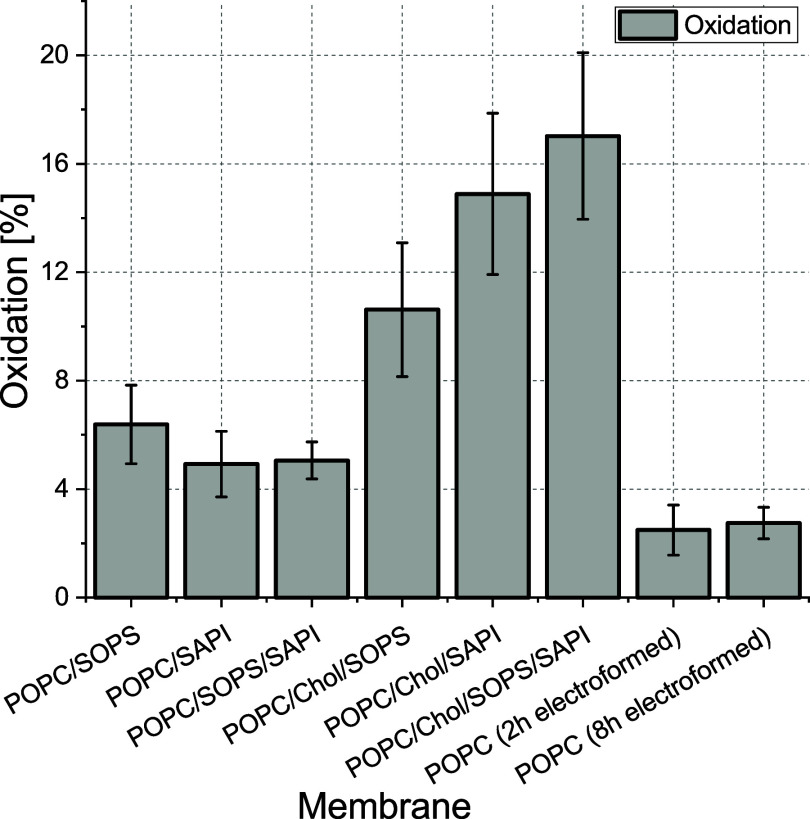
Obtained oxidation levels for investigated membrane compositions in GUVs samples. At least 4 individually prepared populations of GUVs were used to determined the average oxidation level.

### 3.3 Correlation of In Silico and In Vitro Results

To investigate whether the bending rigidity coefficient could be used to estimate the oxidation level in the sample, several steps need to be performed. First of all, a model of bending rigidity coefficient as a function of oxidation needs to be established for each membrane composition. To this end, the data from MD is used as it has the precisely defined oxidation level. To address the issue of limited oxidation points, additional simulation was performed either with 5% or 20%, depending on the experimental result (membrane properties of those systems are presented in Table S4). However, the model of the bending rigidity change as a function of oxidation was assumed to be linear. Such a model is shown in Figure 3A for POPC and Figure 3B for POPC/Chol/SOPS/SAPI mixtures (black dashed line). Then, the bending rigidity value obtained from the experiment was used to reversely calculate the oxidation level in the sample (red dashed lines on the graph) based on the previously established model. Finally, the experimentally determined oxidation level and bending rigidity values including respective uncertainties were also included in the graph (green point). Such an approach was performed for each lipid mixture. The comparison between experimentally determined oxidation and one estimated from bending rigidity is presented in Figure 3C. It should be noted that for all investigated systems, the values are similar. However, due to either the small change in bending rigidity (the case of POPC/SOPS/SAPI—see Figure S5) or high uncertainty value of bending rigidity determination (the case of POPC), the uncertainty of estimation is higher than the value. Nevertheless, the correlation is present for lipid mixtures with significant change in bending rigidity after oxidation such as POPC/Chol/SAPI and POPC/Chol/SOPS, though the accuracy of such an approach is its shortcoming.

**Figure 3:**
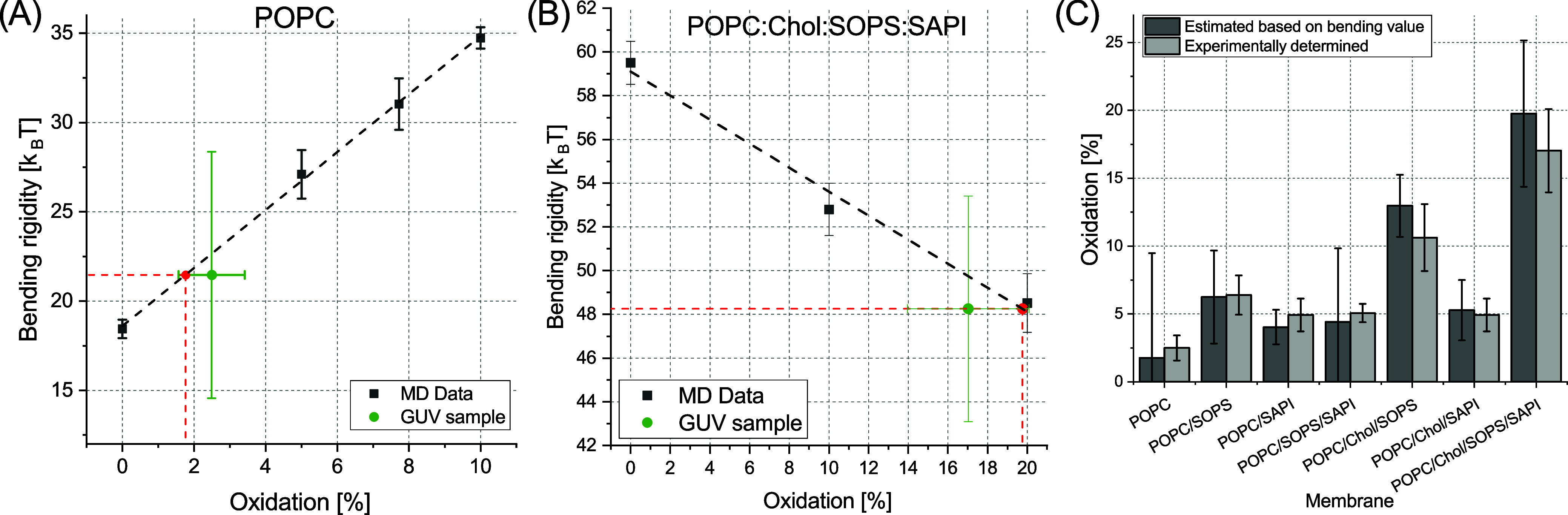
Correlation of computational and experimental approaches. In panels (A,B), results for POPC and POPC/Chol/SOPS/SAPI systems are presented. Black line represents the model of the bending rigidity change as a function of oxidation based on computational results. Green dot represents the experimentally determined bending rigidity and oxidation level, together with uncertainties. Red dot represents the estimation of oxidation level calculated from the model (black line) when experimental bending rigidity is taken as input. (C) Comparison of oxidation levels for each system determined experimentally and calculated based on models determined for each of systems.

These results show that even in a controlled environment, the oxidation is present and should be taken into account. The biophysical properties of membranes are strongly affected by the oxidation process, though depending on the lipid mixture. While the general trend of the oxidation on membrane parameters was not observed, each membrane system had its own specific trend unique to the given lipid mixture. Finally, the change in the bending rigidity is linked to the oxidation of the sample and can be a factor contributing to deviations between the simulation and the experiment.

## 4 Conclusions

The series of simulations and experiments conducted in this study were aimed at investigating the effect of oxidation on more complex lipid mixtures. The lipid mixture was based on lipidomic results of the neuronal membrane. Several findings were made.

The simulation study showed that all of the biophysical properties were affected by oxidation. However, the change in parameters was, in most cases, specific, depending on the mixture. Only in the case of membrane thickness and area compressibility (with the exception for POPC), there was a constant negative change for all of investigated samples. In the rest of the investigated membrane properties, namely, area per lipid, bending rigidity, tilt rigidity, and lateral diffusion, the change varied depending on the sample. This led to the conclusion that each oxidation product can have a different effect over membrane properties.

The experiments revealed that the bending rigidities of the investigated mixtures are, to some degree, in agreement with the range of values obtained for the simulation. However, small deviations were observed. Furthermore, determination of oxidation level in experimental samples showed 2–8% oxidation in mixtures composed only from lipids and 8–20% oxidation in mixtures with additional cholesterol. The reason for the higher oxidation in mixtures with cholesterol is unclear. Finally, it was shown that deviations in experimentally determined bending rigidity can be, to some extent, explained by the oxidation.

Overall, this study provides valuable insights into the alteration of membrane biophysical properties following oxidation and highlights the necessity of investigating oxidative stress within increasingly complex lipid environments. The results indicate that oxidative degradation is a critical factor that must be taken into account when interpreting data from lipid membrane studies. It should be noted, however, that the lipid model employed here was intentionally simplified to prioritize headgroup diversity while maintaining a manageable number of lipid species for systematic analysis.

The most significant of these boundary conditions was the reduction of the polyunsaturated fatty acid (PUFA) fraction. The 5% SAPI content utilized in this study does not fully represent the high PUFA percentages that are characteristic of native neuronal membranes. Consequently, a promising direction for future research would be to incorporate higher PUFA fractions and systematically investigate the biophysical consequences of chain truncation at various carbon positions along the aliphatic tails. See citations from Supporting Information.^53,54^

## Supplementary Material



## Notes

Structures of lipids, details of molecular dynamic systems, detailed numerical values of membrane properties and additional visualizations, details of oxidized lipid parametrization, technical details of experimental oxidation level determination, and correlation between experimental and numerical data for remaining systems (PDF)
