# Supplementary material for: Lipid Oxidation Effect on Membrane Properties in Phosphatidylinositol- and Phosphatidylserine-Enriched Neuronal Biomimetic Models
Source: J Phys Chem B. 2026 Jul 23;130(31):7901–8. doi: 10.1021/acs.jpcb.6c00805 (PMC13455061; doi:10.1021/acs.jpcb.6c00805)
Supplement: Supplementary file 1 [file jp6c00805_si_001.pdf]

Supplementary Information: Lipid Oxidation Effect on Membrane Properties in Phosphatidylinositol- and Phosphatidylserine-enriched Neuronal Biomimetic Model

Dominik Drabik[a,b]

[a] Department of Biomedical Engineering, Wroclaw University of Science and Technology, Wroclaw 50-370, Poland;
[b] Department of Cytobiochemistry, Faculty of Biotechnology, University of Wroclaw , Wroclaw 50-383, Poland;

1. Structures of lipids

The structures of lipids and their truncated counterparts are presented in Figure S1.

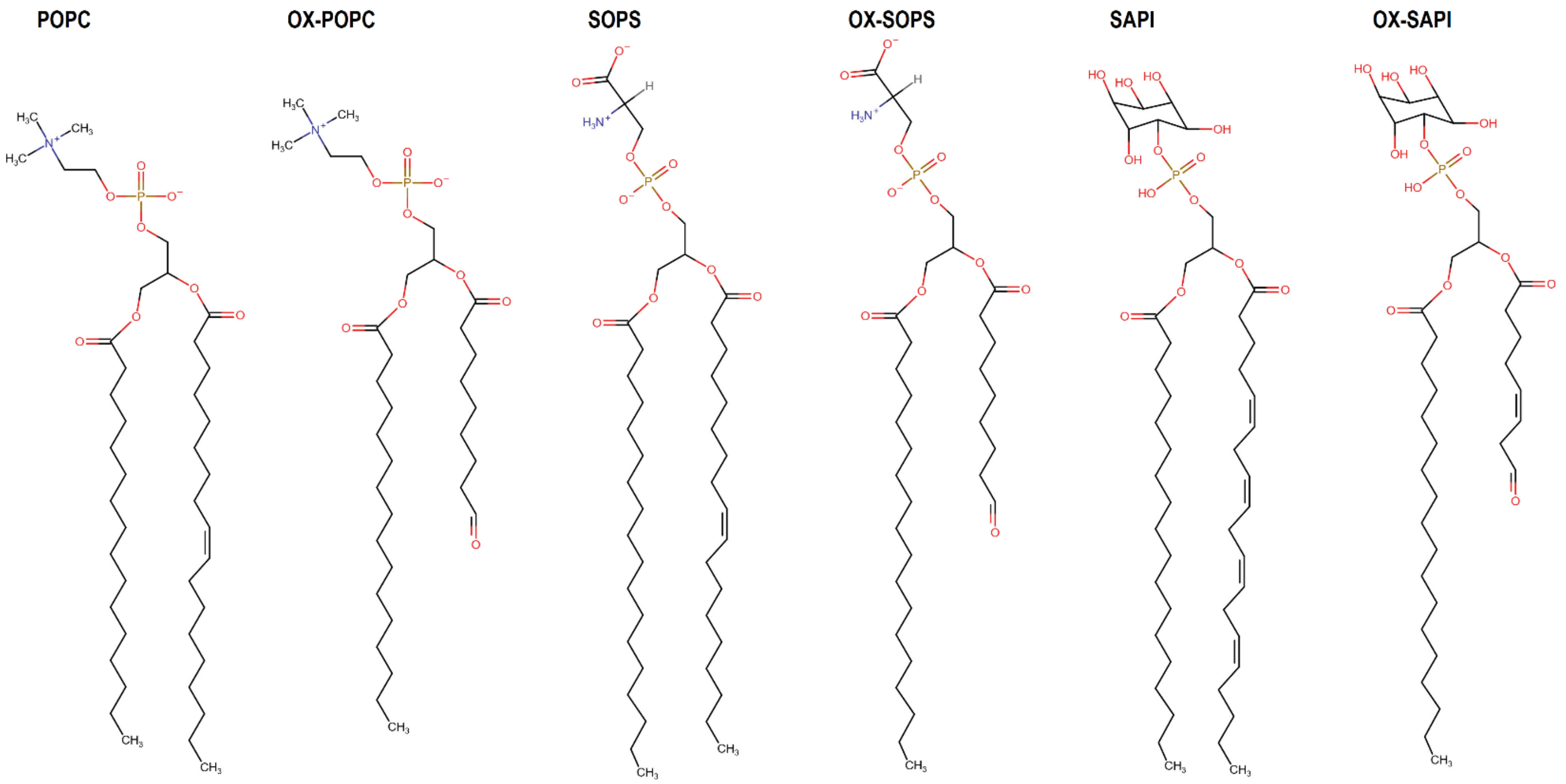

Figure S1. Chemical structures of lipids and their truncated counterparts used in the study.

2. Molecular Dynamics study

The membrane composition was modeled after the human neuronal plasma inner membrane as demonstrated by Ingólfsson *et al* [1], specifically the PC:PE:SM:PS:PI:PA:Chol (14:22:2:10:5:1:46) ratio. To ensure a manageable yet representative model for both computational and experimental analysis, several systematic simplifications were implemented. First, minor components representing the lowest abundance in the native membrane - specifically sphingomyelin (SM) and phosphatidic acid (PA) - were excluded to reduce complexity of the system. Second, phosphatidylethanolamine (PE) was substituted with phosphatidylcholine (PC). This substitution was justified by the fact that PC/PE interactions are extensively documented in existing literature; by consolidating these fractions into a single zwitterionic PC component, it was possible to focus more precisely on the specific influences of PS PI and cholesterol. Consequently, a simplified PC:PS:PI:Chol (38:10:5:47) system was established as the primary model. This molar ratio was strictly maintained across all sub-systems to ensure comparability; for instance, when investigating a binary PC:PI mixture, the 38:5 ratio was preserved (normalized to 88:12).

To reflect the physiological diversity of acyl chain lengths in neuronal bilayers and partial charges, the following lipids were utilized: 16:0-18:1 PC, 18:0-18:1 PS 18:0-20:4 PI. The inclusion of SAPI is of particular importance; as a polyunsaturated fatty acid (PUFA), it serves as a sensitive target for lipid peroxidation, allowing for a robust investigation into how oxidative damage alters membrane mechanics. The details of each system is presented in Table S1. The lipids were randomly selected for oxidation and had their acyl chain truncated. Membrane characterization is presented in Table S2. The Voronoi graphs used for APL determination are presented in Figure S2.

Table S1. Number of lipid molecules used for system composition of investigated MD systems. The number in brackets represents the amount of lipids that were oxidized.

System	POPC	SOPS	SAPI	Cholesterol
POPC/SOPS	518	130	-	-
POPC/SAPI	570	-	78	-
POPC/SOPS/SAPI	460	124	64	-
POPC/Chol/SOPS	260	64	-	324
POPC/Chol/SAPI	272	-	40	336
POPC/Chol/SOPS/SAPI	246	64	34	304
POPC (OX)	648 (64)	-	-	-
POPC/SOPS (OX)	518 (51)	130 (13)	-	-
POPC/SAPI (OX)	570 (58)	-	78 (6)	-
POPC/SOPS/SAPI (OX)	460 (49)	124 (6)	64 (9)	-
POPC/Chol/SOPS (OX)	260 (50)	64 (14)	-	324
POPC/Chol/SAPI (OX)	272 (56)	-	40 (8)	336
POPC/Chol/SOPS/SAPI (OX)	246 (46)	64 (12)	34 (6)	304

Table S2. Membrane properties obtained from molecular dynamic simulations. Values for POPC membrane were taken from [2, 3].

Membrane	MT_{P-P} [nm]	APL [Å^2]	Bending κ [10^{-19} J]	Tilt κ_{tilt} [10^{-20} J]	K_A [mN/m]	Diffusion 2D [μm^2/s]	Interdigitation [nm]
POPC/SOPS	3.98 ± 0.01	60.9 ± 0.2	1.17 ± 0.06	3.32 ± 0.01	535 ± 51	11.1 ± 0.5	0.54 ± 0.01
POPC/SAPI	3.90 ± 0.01	62.4 ± 0.3	1.42 ± 0.06	3.4 ± 0.3	393 ± 26	16.5 ± 0.7	0.52 ± 0.05
POPC/SOPS/SAPI	3.96 ± 0.01	64.9 ± 4.5	1.15 ± 0.02	3.4 ± 0.1	393 ± 57	11.9 ± 0.2	0.35 ± 0.01
POPC/Chol/SOPS	4.40 ± 0.29	39.7 ± 0.6	2.57 ± 0.52	11.9 ± 0.4	9244 ± 203	4.8 ± 0.5	0.39 ± 0.01
POPC/Chol/SAPI	4.17 ± 0.17	38.9 ± 0.2	3.01 ± 0.37	14.0 ± 2.3	9284 ± 659	4.1 ± 0.3	0.27 ± 0.03
POPC/Chol/SOPS/SAPI	4.05 ± 0.01	40.4 ± 0.3	2.44 ± 0.04	12.9 ± 0.5	7994 ± 165	5.86 ± 0.05	0.38 ± 0.01
(OX) POPC	3.74 ± 0.01	63.1 ± 0.2	1.41 ± 0.01	2.97 ± 0.14	230 ± 64	13.44 ± 0.04	0.63 ± 0.03
(OX) POPC/SOPS	3.48 ± 0.03	70.0 ± 1.1	1.18 ± 0.11	3.2 ± 0.3	103 ± 6	15.0 ± 0.9	0.51 ± 0.04
(OX) POPC/SAPI	3.76 ± 0.03	63.0 ± 0.8	1.04 ± 0.11	2.9 ± 0.1	102 ± 6	10.3 ± 1.1	1.02 ± 0.05
(OX) POPC/SOPS/SAPI	3.85 ± 0.03	62.4 ± 1.1	1.08 ± 0.06	3.0 ± 0.3	259 ± 9	11.2 ± 0.7	0.67 ± 0.07
(OX) POPC/Chol/SOPS	3.90 ± 0.08	38.7 ± 0.4	2.42 ± 0.22	11.1 ± 0.9	1558 ± 325	5.28 ± 0.51	0.45 ± 0.05
(OX) POPC/Chol/SAPI	3.95 ± 0.08	38.1 ± 0.2	2.11 ± 0.03	8.6 ± 1.0	1751 ± 15	3.44 ± 0.05	0.37 ± 0.04
(OX) POPC/Chol/SOPS/SAPI	4.12 ± 0.08	38.5 ± 1.2	2.18 ± 0.05	1.01 ± 0.03	1422 ± 73	6.05 ± 0.23	0.41 ± 0.05
POPC (*external data*)	3.92 ± 0.01	63.9 ± 0.2	1.20 ± 0.02	3.42 ± 0.04	235 ± 52	8.31 ± 0.14	0.54 ± 0.06

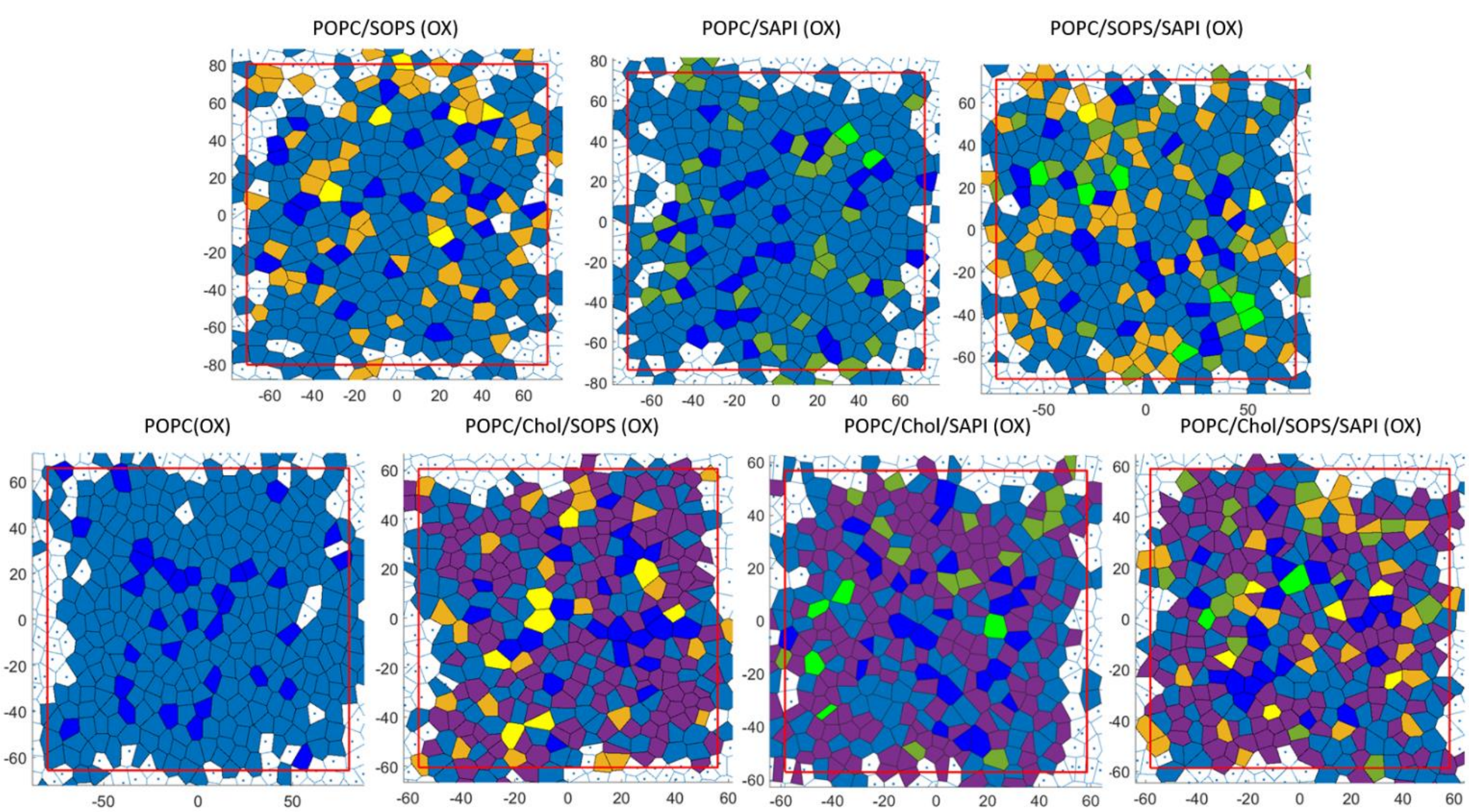

Figure S2. Snapshots of Voronoi analysis for upper leaflet in investigated oxidized systems. Colors represents given lipid type: blue – POPC, dark blue – oxPC, orange – SOPS, yellow – oxPS, olive – SAPI, green – oxPI, violet – cholesterol.

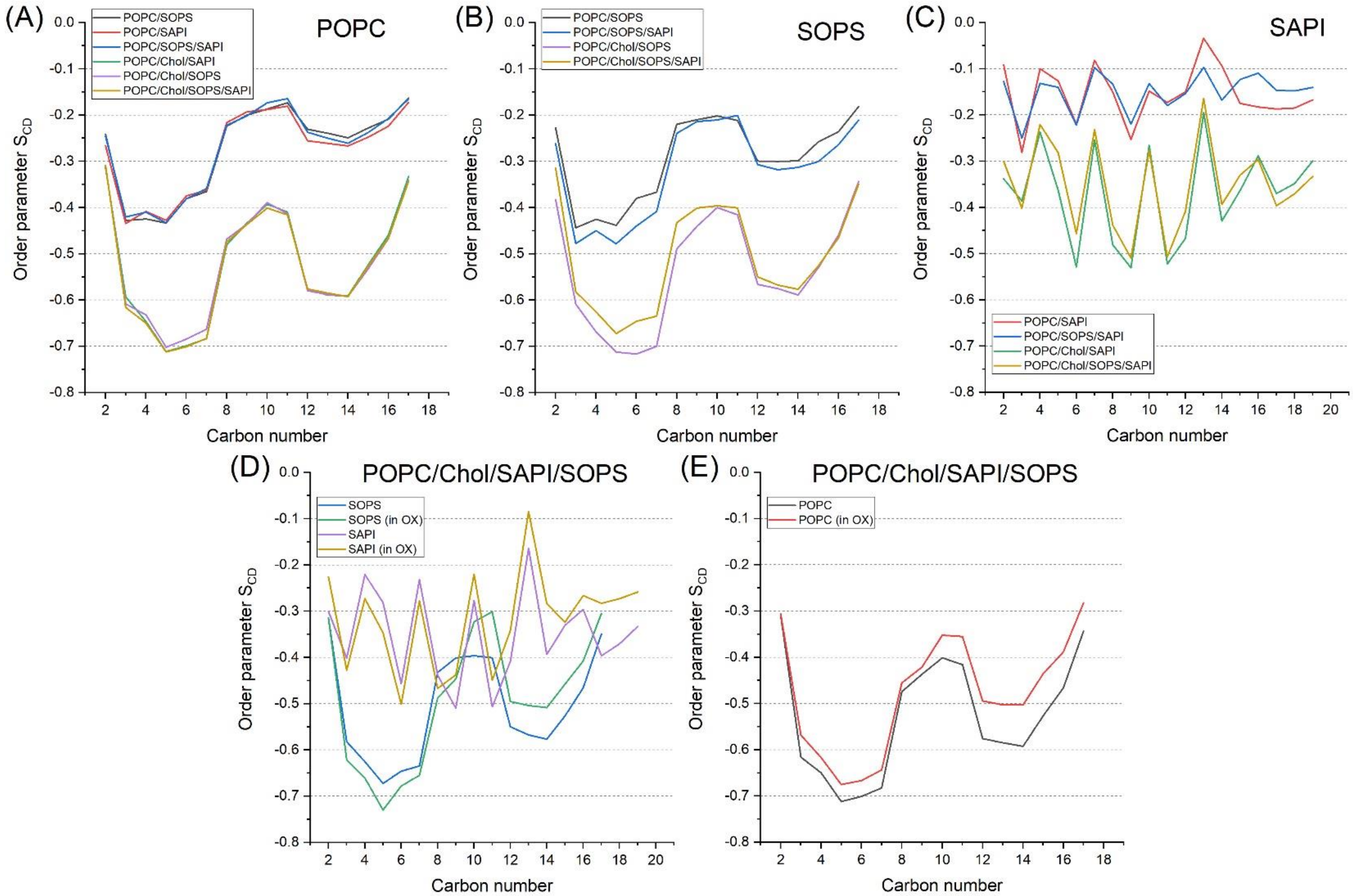

Figure S3. Order parameter for (A) POPC, (B) SOPC and (C) SAPI in different systems prior to oxidation. (D) Change in order parameter for SOPS and SAPI and (E) POPC in POPC/Chol/SAPI/SOPS systems with and without 10% oxidation.

3. OX-lipids parametrization

All three lipids (POPC, SOPS and SAPI) were initially parametrized with CHARMM General Force Field (CGenFF, version 2.5.1) [4]. For OX-POPC the parameter penalty was 0.9 and charge penalty was 0.579, which is within acceptable range. For OX-SOPS the parameter penalty was 14.5 and charge penalty was 8.568 which is within *caution* region, nevertheless, it was used for proceeding with simulations. Finally, in the case of OX-SAPI the parameter penalty was 46.0 and charge penalty was 27.46, which was too high to proceed with the use of this parameterization. To this end FFTK protocol was used for OX-SAPI for calculation of both charges and internal geometry that had high penalty [5]. Briefly, geometry was optimized at MP2/6-31G* level of theory. Charges were optimized with water interaction site approach at HF/6-31G* level of theory, which was followed by optimizing partial atomic charges at MP2/6-31G* level of theory. Bonds and angles were optimized by computing the Hessian at MP2/6-31G* level of theory. Finally dihedrals were optimized first by computing the target data from MP2/6-31g(d) level QM and later optimizing using NAMD software.

4. Oxidation level determination

Oxidation level was calculated as follows: each sample is divided into two halves. One is used for determination of phosphorus amount (which corresponds to lipid molecules) and the second for MDA amount (which is a maker for lipid peroxidation). Calibration curves and results for individual samples are presented in Figure S3. The oxidation level is calculated as n_{MDA}/n_P. At least 4 individually prepared populations of GUVs were used to determined average oxidation level. Results for investigated samples as well as experimentally determined bending rigidity is presented in Table S3.

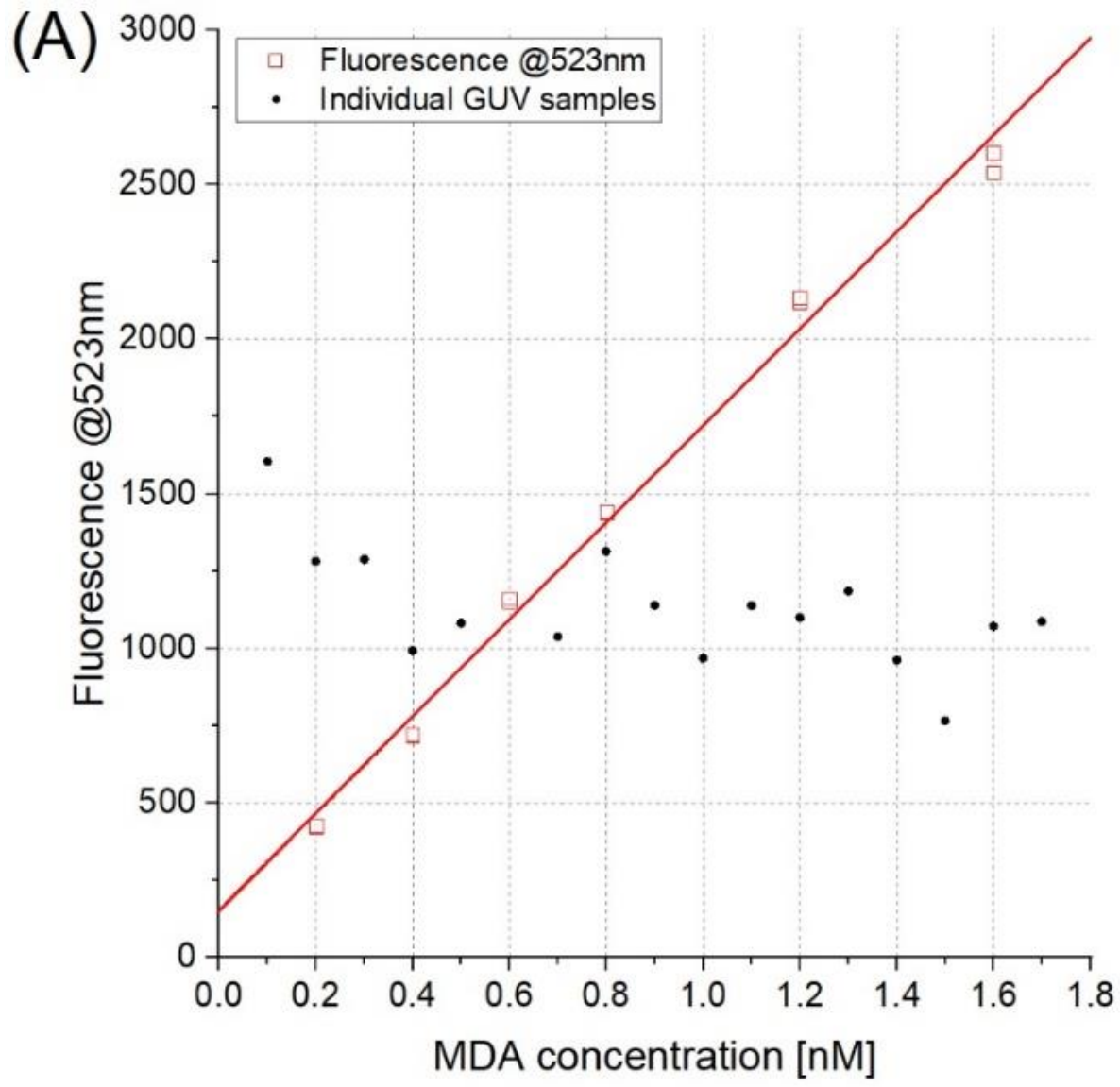

Figure S4. (A) Oxidation calibration curve and (B) phosphor calibration curve along selected individual samples. Individual samples (black dots) presented on the graph are (from left to right): 4x POPC 2h electroformation, 4x POPC 8h electroformation, 2x POPC:SOPS, 2x POPC:Chol:SOPS, 2x POPC:SAPI, 2x POPC:Chol:SAPI.

Table S3. Bending rigidity and oxidation level obtained experimentally for investigated systems.

System	Bending rigidity [k_BT]	Oxidation level [%]
POPC (2h electroformation)	-	2.49 ± 0.01
POPC (8h electroformation)	-	2.75 ± 0.01
POPC/SOPS	87 ± 18	6.39 ± 0.02
POPC/Chol/SOPS	469 ± 54	10.62 ± 0.01
POPC/SAPI	135 ± 26	4.92 ± 0.01
POPC/Chol/SAPI	178 ± 42	14.89 ± 0.03
POPC/SOPS/SAPI	51 ± 14	5.06 ± 0.01
POPC/Chol/SOPS/SAPI	371 ± 66	17.03 ± 0.03

5. Correlation of in-silico and in-vitro results

Table S4. Membrane properties obtained for additional oxidation level using molecular dynamic simulations.

Membrane	Oxidation level [%]	MT_{P-P} [nm]	APL [Å^2]	Bending κ [10^{-19} J]	Tilt $κ_{tilt}$ [10^{-20} J]	K_A [mN/m]
(OX) POPC	5	3.80 ± 0.01	62.9 ± 0.2	1.22 ± 0.05	3.04 ± 0.09	462 ± 13
(OX) POPC	7.7	3.77 ± 0.01	62.8 ± 0.6	1.29 ± 0.07	3.05 ± 0.03	553 ± 37
(OX) POPC/SOPS	5	3.90 ± 0.01	61.6 ± 0.2	1.23 ± 0.03	3.26 ± 0.05	458 ± 48
(OX) POPC/SAPI	5	3.84 ± 0.01	63.1 ± 0.5	1.26 ± 0.09	3.21 ± 0.27	262 ± 103
(OX) POPC/SOPS/SAPI	5	3.90 ± 0.01	61.9 ± 0.3	1.17 ± 0.04	3.12 ± 0.06	242 ± 24
(OX) POPC/Chol/SOPS	20	3.76 ± 0.02	38.1 ± 0.3	2.09 ± 0.03	7.97 ± 0.05	1401 ± 31
(OX) POPC/Chol/SAPI	5	3.91 ± 0.01	38.4 ± 0.4	2.37 ± 0.07	11.9 ± 0.3	1749 ± 151
(OX) POPC/Chol/SOPS/SAPI	20	3.78 ± 0.01	39.6 ± 0.3	2.14 ± 0.02	8.08 ± 0.22	916 ± 14

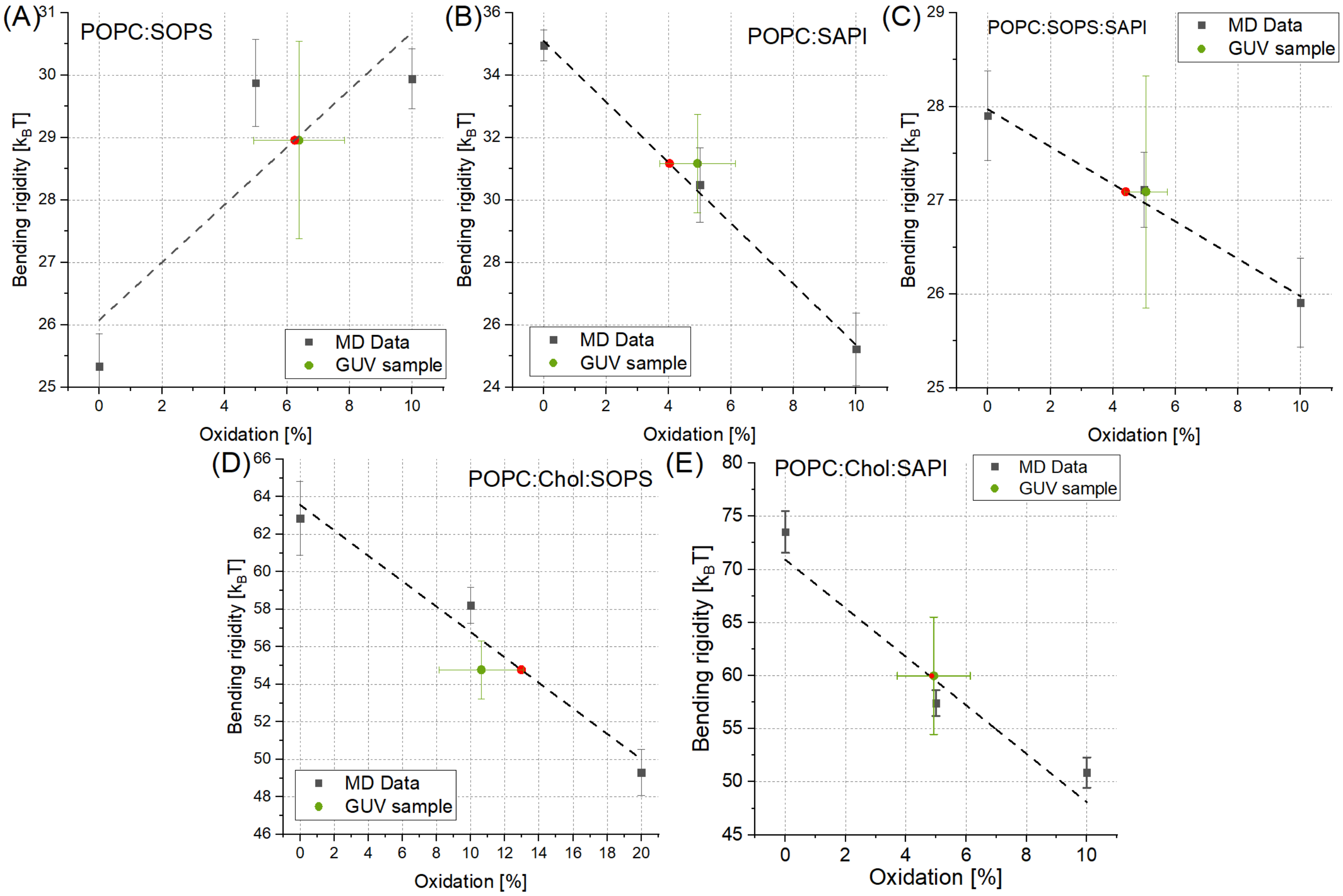

Figure S5. Correlation of computational and experimental approach. Black line represent the model of bending rigidity change in function of oxidation based on computational results. Green dot represent the experimentally determined bending rigidity and oxidation level, together with uncertainties. Red dot represents the estimation of oxidation level calculated from the model (black line) when experimental bending rigidity is taken as input. The results are for following systems: (A) POPC/SOPS, (B) POPC/SAPI, (C) POPC/SOPS/SAPI, (D) POPC/Chol/SOPS, (E) POPC/Chol/SOPS/SAPI.

References

1. Ingolfsson, H.I., et al., *Computational Lipidomics of the Neuronal Plasma Membrane.* Biophys J, 2017. **113**(10): p. 2271-2280.
2. Rzycki, M. and D. Drabik, *Multifaceted Activity of Fabimycin: Insights from Molecular Dynamics Studies on Bacterial Membrane Models.* J Chem Inf Model, 2024. **64**(10): p. 4204-4217.
3. Drabik, D., et al., *Mechanical Properties Determination of DMPC, DPPC, DSPC, and HSPC Solid-Ordered Bilayers.* Langmuir, 2020. **36**(14): p. 3826-3835.
4. Park, S.J., et al., *CHARMM-GUI PDB Manipulator: Various PDB Structural Modifications for Biom-olecular Modeling and Simulation.* Journal of Molecular Biology, 2023. **435**(14).
5. Mayne, C.G., et al., *Rapid parameterization of small molecules using the Force Field Toolkit.* J Comput Chem, 2013. **34**(32): p. 2757-70.
